# Tailoring spintronic and opto-electronic characteristics of bilayer AlN through MnO_*x*_ clusters intercalation; an *ab initio* study

**DOI:** 10.1039/d1ra01532j

**Published:** 2021-04-22

**Authors:** Irfan Ahmed, Yong Shuai, Muhammad Rafique, Mukhtiar Ahmed Mahar, Abdul Sattar Larik

**Affiliations:** Mehran University of Engineering and Technology, SZAB Campus, Khairpur Mirs' Pakistan; School of Energy Science and Engineering, Harbin Institute of Technology 92 West Dazhi Street Harbin 150001 PR China rafique@hit.edu.cn shuaiyong@hit.edu.cn; Mehran University of Engineering and Technology Jamshoro Sindh Pakistan

## Abstract

Adopting *ab initio* density functional theory (DFT) technique, the spintronic and opto-electronic characteristics of MnO_*x*_ (*i.e.*, Mn, MnO, MnO_2_, MnO_3_ and MnO_4_) clusters intercalated bilayer AlN (BL/AlN) systems are investigated in this paper. In terms of electron transfer, charge transfer occurs from BL/AlN to the MnO_*x*_ clusters. MnO_*x*_ clusters intercalation induces magnetic behavior in the non-magnetic AlN system. The splitting of electronic bands occurs, thus producing spintronic trends in the electronic structure of BL/AlN system. Further, MnO_*x*_ intercalation converts insulating BL/AlN to a half metal/semiconductor material during spin up/down bands depending upon the type of impurity cluster present in its lattice. For instance, Mn, MnO and MnO_2_ intercalation in BL/AlN produces a half metallic BL/AlN system as surface states are available at the Fermi Energy (*E*_F_) level for spin up and down band channels, accordingly. Whereas, MnO_3_ and MnO_4_ intercalation produces a conducting BL/AlN system having a 0.5 eV and 0.6 eV band gap during the spin down band channel, respectively. During spin up band channels these systems behave as semiconductors with band gaps of 1.4 eV and 1.2 eV, respectively. In terms of optical characteristics (*i.e.*, absorption coefficient, reflectivity and energy loss spectrum (ELS)), it was found that MnO_*x*_ intercalation improves the absorption spectrum in the low electron energy range and absorption peaks are observed in the 0–3 eV energy range, which are not present in the absorption spectrum of pure BL/AlN. The static reflectivity parameter of BL/AlN is increased after MnO_*x*_ intercalation and the ELS parameter obtains significant peak intensities in the 0–2 eV energy range, whereas for pure BL/AlN, ELS contains negligible value in this energy range. Outcomes of this study indicate that, MnO_*x*_ clusters intercalation in BL/AlN is a suitable technique to tailor its spintronic and opto-electronic trends. Thus, experimental investigation can be carried out on the systems discussed in this work, so as to fabricate practical layered AlN systems that are functional in the field of nano-technology.

## Abstract 2

Atomic structure of MnO_*x*_ cluster intercalated bilayer AlN systems (side view), showing N–Al bond length and interlayer distance in Å, accordingly.

## Graphical abstract

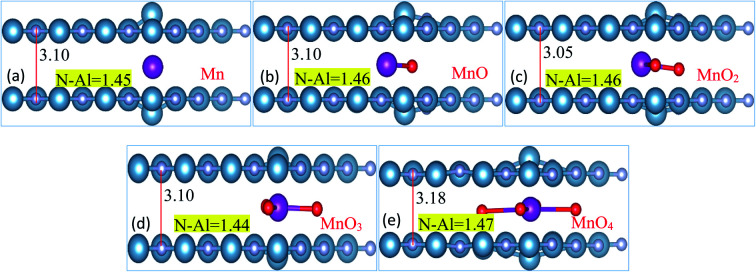

## 1. Introduction

Successful extraction of monatomic two-dimensional (2D) graphene in 2004,^1^ ignited the scientific community to discover more about 2D materials. As a result, various monatomic 2D materials such as, h-BN,^2^ MoS_2_,^3^ SiC^4^ and ZnO^5^ were studied and analyzed experimentally in their monatomic state. These materials were further adopted and investigated for their layered configurations, such as bilayer and few-layer graphene^6^ and h-BN material^7–9^ in order to make them functional for nano-device applications. Further into the 2D materials family, group III-Nitride 2D materials carry significant potential for their use as state of the art solid state devices, sensors/actuators and visible range photo-catalysis process.^10–13^ Among these group III-Nitrides, monatomic AlN with a 3.92 eV band gap^14^ offers promising applications as a DMS (dilute magnetic semiconductor). Theoretical investigations based on DFT technique carried out on the DMS behavior of the monatomic AlN layer indicated that, proper/selective substitution of transition metal (TM) atoms in the AlN layer can make it display ferromagnetic behavior above room temperature.^15–18^ Electronic properties of AlN nanotubes and nanowires were studied by Zhou *et al.*,^19^ and the authors suggested AlN nano-tubes and wires can be utilized for ammonia sensing applications. Similarly, nano-ribbons of pristine AlN were synthesized by Xie *et al.*^20^ using a chloride-assisted-vapor-solid-route technique.

From aforementioned studies it can be realized that, significant work is already being performed on monolayer and nano-tubes of AlN system. However, bilayer or multilayered AlN systems are slightly studied. Consequently, conception of interlayer interference and the properties of layered AlN systems is a thought to discover and it can produce appealing progress in the field of layered AlN systems. Earlier, significant efforts have been carried out to understand the structural, electronic and energetic of single and multi-layered h-BN systems.^21–26^ Various concepts to be understood for multi-layered systems include, Moiré patterns upon relative rotation of few layers^27,28^ and observations of displacement in stacked layers.^29–31^ Experimental studies carried out on impurity clusters intercalated layered graphene systems,^32–34^ suggest that, foreign intercalated impurities can significantly modify the opto-electronic and magnetic parameters of layered systems. Similarly, T. Kaneko *et al.*^35^ carried out first-principles investigation on alkali and alkaline earth metal intercalated bilayer graphene systems, indicating that these intercalated atoms can greatly modify its energetic and conductivity parameters. In addition, very recently, studies have been carried out on layered 2D materials having defective configurations.^36–40^ These, theoretical and experimental studies conclude that physical parameters of layered 2D systems can greatly be modified through intercalation technique. All the earlier studies carried out on layered 2D systems suggest that, electronic, magnetic and optical behaviors of these stacked systems can be manipulated by modifying their stacking position. Though, another suitable approach to adapt these parameters is through chemical doping process. Impurity atoms/clusters can be substituted or intercalated in the layered systems, thus producing a hybrid material. For instance, experimental studies were performed on impurity clusters intercalated few layered graphene systems.^32–34^ Outcomes of these studies revealed that, foreign impurities when doped/intercalated into layered systems can easily alter the electronic, magnetic and optical parameters of layered 2D systems as compared to their pristine counterparts. Likewise, H. Jinsen *et al.*^41^ performed *ab initio* DFT calculations on TM atoms intercalated bilayer graphene and it was found that, TM intercalation in bilayer graphene can produce a stable 2D magnetic substrate. Few studies carried out on twisted/defective h-BN layers^42–45^ suggest that, homo/hetero/defective layers can produce functional layered materials for nano-electronic and energy storage applications.

Since, significant amount of work has been dedicated to few layered graphene and h-BN systems, so this concept can be elongated for AlN layers, in order to obtain a functional layered AlN system. Akin to monatomic AlN, bilayer (BL)/AlN is also an insulating material, with non-magnetic nature and behaves opaquely in the visible range of spectrum.^7^ Since, BL/AlN is a wide band insulator; hence it cannot be utilized in fields of energy storage or smart generation applications. So as to make it functional for energy storage, optoelectronic and spintronic device applications, it is essential to modify its physical behaviors first. In order to gain aforementioned objectives, MnO_*x*_ clusters were adopted as impurities and were intercalated in BL/AlN system. The effect of MnO_*x*_ intercalation on the structural, electronic, magnetic and optical properties of BL/AlN systems was investigated in detailed manner. MnO_*x*_ clusters were adopted as intercalation agents owing to their excess of charge and easy transport nature. MnO_*x*_ clusters can behave as n-type or p-type impurities, depending upon the layered configurations; thereby manipulating the band gap in the electronic structure of BL/AlN system. It is well known fact that, optical transitions are linked to the inter/intra band transitions occurring in the electronic structures, hence modifying band gap will directly affect the optical behaviors of BL/AlN system. Furthermore, regarding magnetic behavior, it can be proposed that the unfilled d orbitals of Mn atom can produce significant spin variation in the BL/AlN, thus producing a magnetic bilayer substrate. As stated in earlier studies that, the halogen and super halogen *i.e.*, TMO_3_ and TMO_4_ clusters can greatly modify the physical parameters of monolayer graphene^46,47^ and h-BN^48^ systems. Hence, we try to extend this notion to BL/AlN systems. Up to our knowledge, such complex systems of AlN material have been lightly touched and comprehension of these systems remains partial and scattered. Obtained outcomes of this work can provide a subtle path for tailoring spintronic and opto-electronic trends of BL/AlN systems to be functional for nano-electronic devices, distinctive to those of pristine monolayer AlN systems.

## 2. Computational details and geometry models

**Fig. 1:**
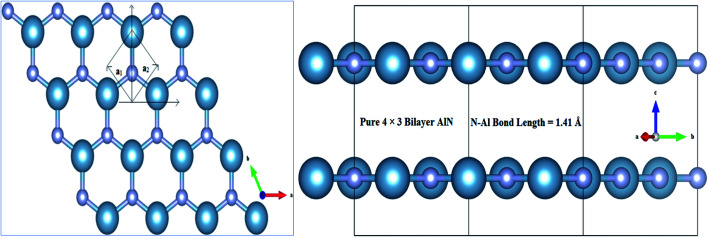
Illustration for atomic structure of 4 × 3 BL/AlN system (a) normal and (b) in-plane view of optimized geometry of BL/AlN system.

Adopting *ab initio* DFT calculations in Generalized Gradient Approximation (GGA)^49–51^ implemented in Vienna *Ab initio* Simulation Package (VASP version 5.2.2),^52–54^ the electronic, magnetic and optical behaviors of MnO_*x*_ (*i.e.*, Mn, MnO, MnO_2_, MnO_3_ and MnO_4_) intercalated BL/AlN were studied in detail. A 4 × 3 supercell structure was adopted for Bilayer AlN (BL/AlN)^55,56^ and the computations were carried out through plane-wave basis set with ultrasoft psuedopotentials^52,57^ having 450 eV cut-off energy. All computations were performed in spin polarized mode. Grimme (DFT-D2)^49^ method was utilized for van der Waals (vdW) corrections due to the long-range vdW interactions in between layers.^58–60^ A 15 Å vacuum thickness was added along the *Z*-direction so as to reduce the interference problem between adjacent layers.^55,61,62^ Larger vacuum thickness size supports omission of interlayer interference. In order to gain satisfactory convergence in the DFT results, a fine 17 × 17 × 1 *k*-point mesh was employed. Geometry relaxation was performed till the Hellmann–Feynman forces were up to 0.03 eV Å^−1^ value and variation in total energy step less than 10^−6^ eV was not achieved. Gaussian smearing was implemented in order to deal with partial occupancy problem. Normal and in-plane views of relaxed geometry of pristine 4 × 3 BL/AlN system are presented in Fig. 1(a) and (b), in that order. The terms 
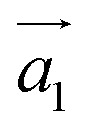
 and 
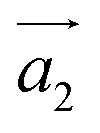
 correspond to the vector quantities of AlN unit cell in the figures given below.

In order to calculate fine grid electronic properties, 30 *K*-points were adapted for *Γ* − *M* − *K* − *Γ* path in the irreducible Brillouin zone (IBZ). For calculating partial/local and total density of states of MnO_*x*_ intercalated BL/AlN systems, 17 × 17 × 1 *k*-points were utilized and a 0.03 eV Gaussians width was employed for eigenvalues smearing. Total/partial DOS on constituents of AlN system and intercalated impurities were determined in spin polarized mode. Subjected to the band gap sensitivity problem, we investigated both the Local Density Approximation (LDA) and Perdew–Burke–Ernzerhof exchange-correlation (PBE) functionals, subsequently, it was observed that the functional choice did not produce larger band gap variation and a slight variation of ∼0.02 eV was observed in the band gap.

Likewise, for optical properties, DFT within Random Phase Approximations (RPA)^63^ technique was implemented. The imaginary part of dielectric constant was obtained through summation of empty states, as described. In eqn (1), α and β are Cartesian vectors, *e*_α_/*e*_β_ are primal vectors and the *c*/*v* parameters depict conduction and valence bands, respectively. The terms *∈*_*ck*_/*∈*_*vk*_ respond to the corresponding energy of *c*/*v* bands, cell periodic part at a given point *k* is denoted *u*_*ck*_ term.1
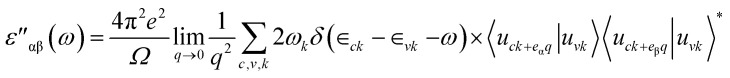


The real part of dielectric constant can be obtained through Kramers–Kronig transformation as described. Here *P* parameter depicts Principal value.^63^2
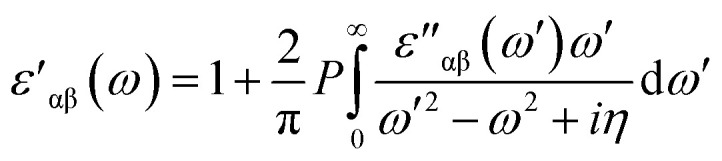


Obtained real (*ε*′) and imaginary (*ε*′′) parts of dielectric constant can be utilized for extracting total dielectric constant by *ε* = *ε*′ + *ε*′′ together.^63^ Later on, optical absorption ‘α’ and reflectivity ‘*R*’, real and imaginary refractive index *i.e.*, ‘*n*’ and ‘*k*’ parameters can be determined through dielectric constant, as explained in detail in earlier reports.^64,65^ For aforementioned optical properties, focus of our work is directed towards lower energy *i.e.*, 0–12 eV energy range. Our main goal is to modify the absorption spectrum of bilayer AlN system in visible range, along with its static reflectivity by MnO_*x*_ intercalation. MnO_*x*_ intercalated BL/AlN systems with modified optical properties in the visible region can be a viable candidate for opto-electronic applications as compared to its single layer counterpart.

## 3. Results and discussions

Outcomes of this study along with their relevant analysis are provided as follows;

### 3.1 Structural properties of MnO_*x*_ intercalated BL/AlN

**Fig. 2:**
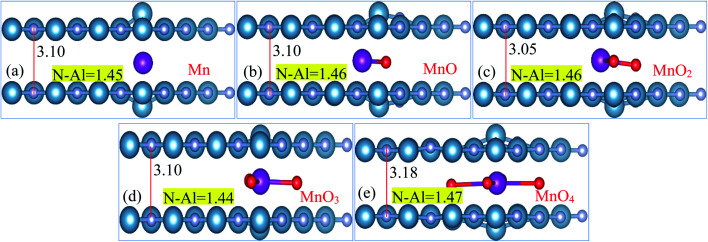
(a)–(e) Atomic Structure of MnO_*x*_ cluster intercalated bilayer AlN systems (side view), showing N–Al bond length and interlayer distance in Å, accordingly.

Structural diagrams of bilayer AlN system intercalated with MnO_*x*_ (*i.e.*, Mn, MnO, MnO_2_, MnO_3_ and MnO_4_) clusters are presented in Fig. 2(a)–(e), respectively. When MnO_*x*_ clusters are intercalated in BL/AlN, these clusters try to hold their position at the center of interlayer space as visible in Fig. 2(a) and (b). However, after MnO_*x*_ cluster intercalation, the bond lengths of N–Al atoms of both layers available in the vicinity of impurities get distorted. Thus a variation in range of 1.44–1.47 Å in N–Al bond lengths is obtained in both AlN layers. Bond length variation observed in N–Al atoms is totally dependent on the type of MnO_*x*_ cluster available in the interlayer space as visible in Fig. 2(a)–(e), respectively. A change in the interlayer distance was also observed ranging in between 3.10–3.18 Å, again totally dependent on the type of MnO_*x*_ cluster intercalated in BL/AlN. These outcomes are consistent with earlier reports.^66,67^

Total magnetic moment of various MnO_*x*_ cluster intercalated BL/AlN systems, magnetic moments of Mn atom and the equatorial bond distances of N–Al, Mn–O and interlayer distance for that particular complex systems are enlisted in Table 1, in that order. Presented accumulative magnetic moments of various MnO_*x*_ cluster intercalated BL/AlN systems depict that, MnO_*x*_ clusters significantly improve the ferromagnetic behavior in BL/AlN as evident by their larger values of magnetic moments, respectively.^46,68^ Some earlier studies carried out on magnetism of 2D materials have suggested the same behaviors.^69–71^ Further, it becomes necessary to determine first whether the given MnO_*x*_ cluster intercalated BL/AlN systems are thermodynamically stable or not, binding energy of each system should be calculated first. Binding energies for given systems can be calculated using following expression^66^ and are enlisted in Table 1,

3 *E*_b_ = *E*_(AlN+MnO*x*)_ − (2*E*_(AlN)_ + *E*_MnO*x*_)

here, the *E*_(AlN)_, *E*_MnO*x*_ and *E*_(AlN+MnO*x*)_ terms correspond to, the total energy of AlN layers, total energy of MnO_*x*_ clusters and the total energy of MnO_*x*_ cluster-incorporated bilayer AlN systems, correspondingly. Obtained binding energies clearly indicate that, MnO_*x*_ cluster intercalation in bilayer AlN systems is thermodynamically stable as mentioned systems have positive binding energies. Obtained results are consistent with earlier reports.^41,66,72,73^

**Table 1:** Total magnetization of the supercell (*μ*_tot_, in *μ*_B_), magnetic moments of individual Mn atom, the binding energies *E*_b_ (eV), bond distances of N–Al (d_N–Al_, in Å), Mn–O (d_Mn–O_ in Å) and interlayer distance AlN–AlN (d_AlN–AlN_ in Å) atoms for all MnO_*x*_ cluster-sandwiched bilayer graphene systems

Impurity	*μ*_tot_, in *μ*_B_	*μ*_MN_, in *μ*_B_	d_N–Al_ Å	d_Mn-O_ Å	d_AlN–AlN_ Å	*E*_b_ (eV)
Mn	3.7	2.835	1.45	2.10	3.10	2.99
MnO	2.76	1.58	1.46	1.98	3.10	3.01
MnO_2_	3.01	2.51	1.46	2.11	3.05	3.36
MnO_3_	1.03	0.96	1.44	1.95	3.10	3.72
MnO_4_	1.12	0.93	1.47	1.98	3.18	4.48

**Fig. 3:**
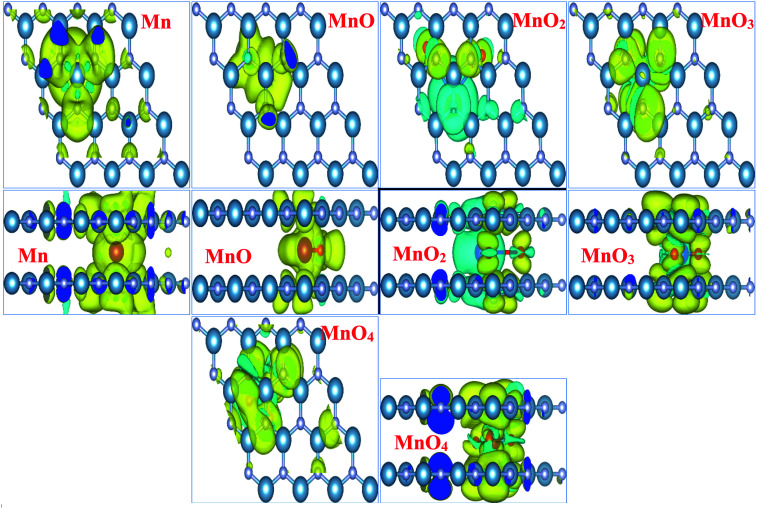
Illustration (top and side views) of spin difference diagrams for MnO_*x*_ cluster-intercalated bilayer AlN systems. Yellow and cyan isosurfaces (0.002 e Å^−3^) depict positive and negative spin of electrons, respectively.

Fig. 3 illustrates the spin density of all the MnO_*x*_ cluster-intercalated bilayer AlN systems. Given Fig. 3 clearly defines that the intercalated MnO_*x*_ clusters create magnetic behavior in non-magnetic N and Al atoms of AlN layer lying above and below the impurity clusters. In addition, spin density created in the bilayer AlN layer is not localized rather it is distributed throughout the bilayer plane as visible in the top view of spin density diagrams. Almost all the MnO_*x*_ clusters and AlN layers attain anti-parallel spin polarization direction as visible in the side view of spin difference diagrams. However, only in case of MnO_2_ intercalation parallel spin polarization is observed in between impurity cluster and Bilayer AlN. Main attribution towards magnetism is by the unfilled d-orbital electrons of Mn atoms carrying clockwise spin direction as evident by yellow isosurface (0.002 e Å^−3^), for all given MnO_*x*_ clusters except MnO_2_ cluster. Similarly, parallel spin polarization was observed between Mn and O atoms except MnO cluster-incorporated BL/AlN, which carried antiparallel spin polarization as shown in Fig. 3, respectively. These observations are persistent with earlier reports.^66,73,74^

**Fig. 4:**
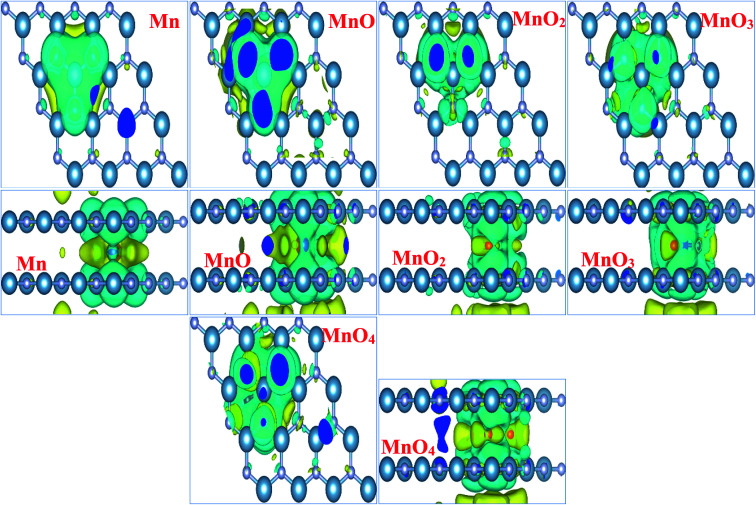
Illustration (top and side views) of charge density difference diagrams of MnO_*x*_ cluster-intercalated bilayer AlN systems. Yellow and cyan isosurfaces (0.0001 e Å^−3^), depict gain and loss of electrons, respectively.

In order to totally comprehend the electronic interaction of MnO_*x*_ clusters with BL/AlN systems, we investigated charge difference and transfer behavior of all MnO_*x*_ cluster-incorporated BL/AlN systems through Bader analysis.^75,76^ The charge difference can be obtained by Δ*ρ* = *ρ*_MnO_*x*_-BL/AlN_ − *ρ*_BL/AlN_ − *ρ*_MnO_*x*_. Where_*ρ*_MnO_*x*_-BL/AlN_, *ρ*_BL/AlN_ and *ρ*_MnO_*x*__ display charge density of MnO_*x*_ cluster-intercalated BL/AlN, charge density of AlN layers and the charge density of MnO_*x*_ clusters, in that order. Obtained charge difference diagrams for all MnO_*x*_ cluster-intercalated BL/AlN systems are offered in Fig. 4, respectively. The yellow (0.0001 e Å^−3^) and cyan isosurfaces (0.0001 e Å^−3^) display the occurrence of electron gain and loss behavior between MnO_*x*_ clusters and BL/AlN, respectively. Illustrated charge difference diagrams of MnO_*x*_ cluster-intercalated BL/AlN complexes carry similar charge transfer phenomenon *i.e.*, AlN layer lose their charge to electronegative MnO_*x*_ clusters as these clusters carry larger chunk of yellow isosurface while AlN layer are covered by cyan isosurface thus indicating charge transfer direction from AlN layers to MnO_*x*_ clusters, respectively. As bilayer AlN systems display electron loss behavior thus MnO_*x*_ intercalation can be considered as “Hole” doping process. These predictions are in consensus with earlier published reports.^77–80^

### 3.2 Electronic structures of MnO_*x*_ cluster-intercalated BL/AlN

**Fig. 5:**
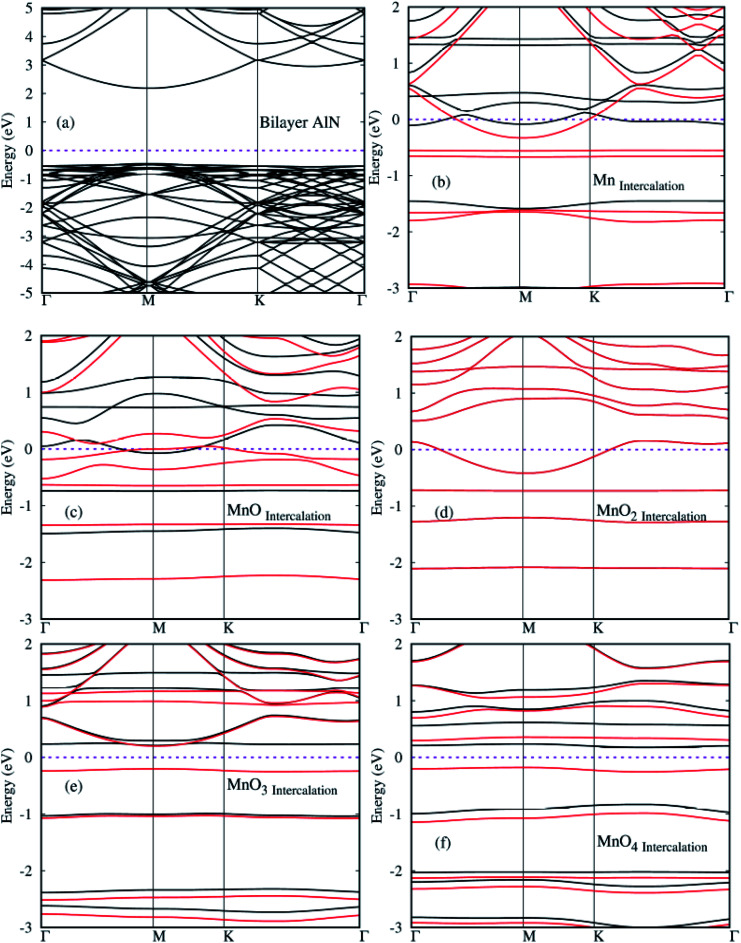
(a)–(f) Spin polarized electronic structures for MnO_*x*_ cluster-intercalated BL/AlN (4 × 3) supercell structures. Black and Red lines depict spin up and spin down bands, respectively.

In this section, the spin polarized electronic structures and density of states (DOS) plots of pure and MnO_*x*_ cluster (*i.e.*, Mn, MnO, MnO_2_, MnO_3_ and MnO_4_) intercalated bilayer AlN complex were analyzed in detail and are illustrated in Fig. 5(a)–(f), respectively. Fermi Energy level (*E*_F_) is shown by purple color dotted line drawn at 0 eV energy in given diagrams. As a reference, the band structure of pure BL/AlN is added as shown in Fig. 5(a) and our obtained work is consistent with these reports,^81–83^ which indicates that our computational method is accurate enough to carry out further calculations in this regard. Band gap value of BL/AlN was found as ∼3.92 eV which agrees well with earlier cited reports. After MnO_*x*_ intercalation in BL/AlN significant change is observed in its electronic structure. Mn, MnO and MnO_2_ intercalation in BL/AlN converts wide band BL/AlN to a half metallic material for both spin up and down bands respectively, as some energy bands appear at the *E*_F_ level as shown in Fig. 5(b)–(d), in that order. However, during MnO_3_ and MnO_4_ intercalation, wide band BL/AlN is converted to narrow band semiconducting material for both spin up and down channels as shown in Fig. 5(e)–(f), respectively. Obtained band gap during MnO_3_ intercalation is found to be 1.2 eV and 0.4 eV, during spin up and spin down band channels, as shown in Fig. 5(e), respectively. Similarly, band gap obtained through MnO_4_ intercalation was 1.3 eV and 0.5 eV, during spin up and spin down band channels, as shown in Fig. 5(f), respectively. As per above discussion, it can be presumed that, MnO_*x*_ intercalation can be a suitable technique to modify electronic structures of BL/AlN systems. Given results are in consensus with previous literature.^84–86^

**Fig. 6:**
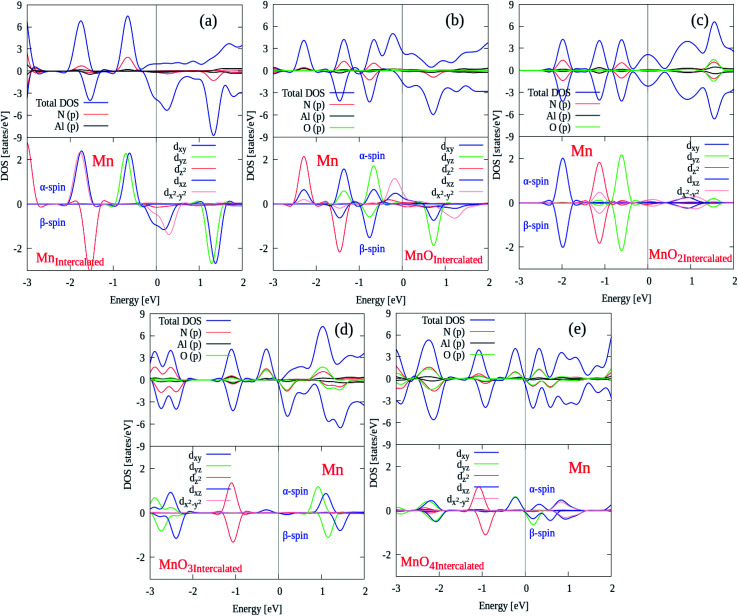
(a)–(e). Description of P/L-DOSs plots for all MnO_*x*_ cluster-intercalated BL/AlN systems.

After electronic structures, the partial and local (P/L DOS) plots for all MnO_*x*_ cluster-incorporated BL/AlN systems are investigated, so that the orbital interaction between impurity cluster atoms and BL/AlN can be analyzed. P/L-DOS on d orbitals of Mn atoms and constituent Al, N and O atoms of MnO_*x*_ BL/AlN were calculated and are produced in Fig. 6(a)–(d), respectively. The *E*_F_ level is depicted at 0 eV energy drawn by vertical grey line in given P/L-DOS plots. As mentioned in Table 1, all the MnO_*x*_ intercalated BL/AlN systems carried definite magnetic moments; hence the d orbitals of Mn atom and the sp orbitals of N, Al and O atoms carried spin polarization thus creating variation in band gap value for spin up and down bands as clearly depicted in Fig. 6(a) and (d), respectively. From the given band structure and DOS plots of MnO_*x*_ intercalated BL/AlN systems it can be generalized that, MnO_*x*_ intercalation in BL/AlN not only tunes the band gap parameter but can convert it to DMS material. Since these systems carry finite magnetic moments and differing band gap values during spin up and down channels. These astonishing properties of MnO_*x*_ intercalated BL/AlN systems can make it practical choice for spintronic devices.

### 3.3 Optical properties of MnO_*x*_ cluster-intercalated BL/AlN

Lastly, we calculate the optical characteristics of MnO_*x*_ clusters (*i.e.*, Mn, MnO, MnO_2_, MnO_3_ and MnO_4_) intercalated BL/AlN systems through RPA-DFT.^63^ In RPA-DFT Local field effects are omitted while inter/intra band transitions are considered, thus inaccuracies can be expected in dielectric constant at lower energies. A fine 11 × 11 × 1 *Γ*-centered BZ sampling is adopted for optical properties calculations.

**Fig. 7:**
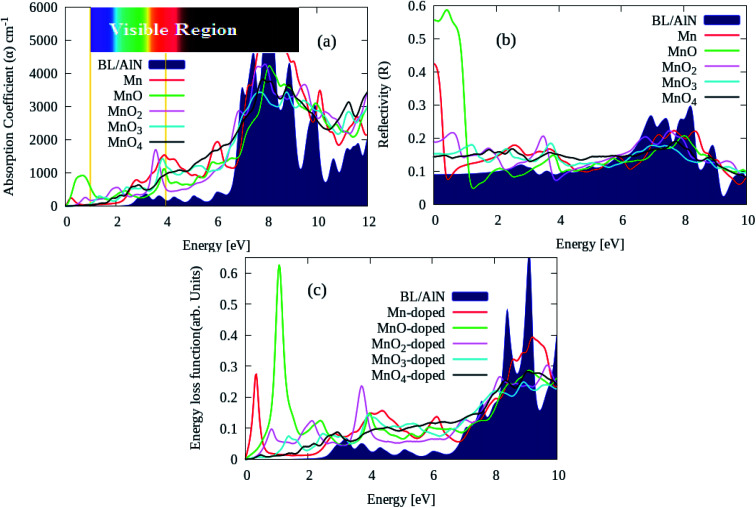
(a) Absorption coefficient ‘α’, (b) reflectivity ‘*R*’ and (c) ELS parameters of pure and MnO_*x*_ clusters intercalated BL/AlN systems, respectively.

Absorption ‘α’, reflectivity ‘*R*’ and energy loss spectrum ‘ELS’ parameters can easily be calculated through dielectric constant as elaborated in ref. 64. The absorption coefficient of pure and various MnO_*x*_ clusters intercalated BL/AlN systems are illustrated in Fig. 7(a). Our main focus is dedicated to tailoring optical parameter of BL/AlN in lower energy *i.e.*, 0–12 eV range. The absorption spectrum of pure BL/AlN has no absorption peaks up to 3 eV energy layer as visible in Fig. 7(a). However, after MnO_*x*_ intercalation, absorption coefficient starts to rise from 0 eV energy. As seen, MnO and MnO_2_ intercalation produces absorption intensity of 1000 cm^−1^ and 2000 cm^−1^ at 0.75 eV and 3.5 eV energy points which were not available in case of pure BL/AlN system. In addition, MnO_*x*_ intercalation overall improves the absorption spectrum of BL/AlN in lower energy as obvious in Fig. 3(a).^87,88^

Likewise, the ‘*R*’ parameter of aforementioned systems is provided in Fig. 7(b). MnO_*x*_ intercalation improves static ‘*R*’ parameter of BL/AlN system having 0.58 maximum peak intensity of MnO intercalated BL/AlN system as revealed in Fig. 7(b). Lastly, ELS parameter of pure and MnO_*x*_ cluster-intercalated BL/AlN systems is shown in Fig. 7(c). It can be observed that, MnO_*x*_ clusters provide larger energy variation to the electrons of BL/AlN system in between 0–2 eV energy as noticeable in Fig. 7(c), accordingly.^88,89^ In general, it can be presumed that, MnO_*x*_ cluster intercalation in BL/AlN can significantly improve its optical parameters in low electron energy range thus making it functional for opto-electronic device applications. From given MnO_*x*_ cluster intercalated in BL/AlN systems, comparatively it can be suggested that MnO-intercalated BL/AlN system bears suitable optical parameters thus, this model can be realized for experimental studies.

## 4. Conclusion

In this study, we analyzed various MnO_*x*_ clusters (*i.e.*, Mn, MnO, MnO_2_, MnO_3_ and MnO_4_) intercalated BL/AlN systems through FPS-DFT technique. As MnO_*x*_ clusters offer higher electronegative nature, thus stable BL/AlN systems containing MnO_*x*_ clusters as impurities can be realized as obvious through obtained positive binding energy values for all systems. Spin difference diagrams of given systems clearly suggest that magnetic BL/AlN systems can be generated through MnO_*x*_ clusters intercalation. Magnetic moments for Mn, MnO, MnO_2_, MnO_3_ and MnO_4_ intercalated BL/AlN systems were obtained as 3.7 μ_B_, 2.76 μ_B_, 3.01 μ_B_, 1.03 μ_B_ and 1.12 μ_B_, respectively. Through charge transfer diagrams it is revealed that, the charge transfer occurs from BL/AlN to MnO_*x*_ clusters, hence creating hole doping process in BL/ALN system.

Through calculated spin polarized band structures, it was observed that the MnO_*x*_ clusters intercalation in BL/AlN, the wide band insulating BL/AlN can be converted to half metal/semiconductor depending upon the type of intercalated MnO_*x*_ cluster. Through Mn, MnO and MnO_2_ intercalation half metallic BL/AlN system is achieved for both spin up and down bands respectively. For MnO_3_ and MnO_4_ intercalation, wide band BL/AlN is converted to narrow band semiconducting material for both spin up and down channels. Obtained band gap during MnO_3_ intercalation is found to be 1.2 eV and 0.4 eV, during spin up and spin down band channels, respectively. Whereas, band gap obtained through MnO_4_ intercalation was 1.3 eV and 0.5 eV, during spin up and spin down band channels, respectively. In addition, through PDOS analysis it is revealed that, the intercalated systems having finite magnetic moments exhibit orbital polarization phenomenon. It is also presumed that the d orbitals (*i.e.* d_*xy*_, d_*yz*_, d_z^2^_, d_*xz*_ and d_*x*^2^–*y*^2^_) of Mn atom are mainly contribute to the obtained magnetic behavior of BL/AlN system. Thus suggesting functionality of studied systems in spintronic devices.

Finally, through optics properties, it is observed that the ‘*α*’ and ‘*R*’ and ELS parameters of MnO_*x*_ intercalated BL/AlN systems achieve significant variation in low electron energy range. In addition, MnO_*x*_ intercalation introduces red shift to ‘α’ parameter and it is significantly improved in visible region. In general conclusion and through detailed analysis, it can be suggested that MnO_*x*_ cluster-intercalated BL/AlN systems demonstrate significant potential for being functional for innovative spintronic and optoelectronic applications, which are distinctive to their pristine BL/ALN systems.

## Conflicts of interest

There are no conflicts to declare.

## Supplementary Material
